# Hispanic Views About Online Information on Weight Gain, Diet, and Physical Activity in Pregnancy: A Qualitative Study

**DOI:** 10.1177/23333936251334514

**Published:** 2025-04-17

**Authors:** Sookja Kang, Lorraine O. Walker, Kelly Pretorius, Michael Mackert

**Affiliations:** 1 The University of Texas at Austin, USA; 2 Texas Children’s Hospital, Austin, USA

**Keywords:** qualitative, gestational weight gain (GWG), diet, physical activity, pregnancy, online health information, Hispanic, Southwest USA

## Abstract

The vast amount of online health resources accessible to the public and the pregnant population shows their high interest in using online health resources for their pregnancies. In this study, we specifically aimed to understand the experience and use of online pregnancy health information among Hispanic individuals, who are at higher risk of gaining outside of the recommended guideline of gestational weight gain (GWG) than the overall U.S. childbearing population. We conducted face-to-face semi-structured interviews with 20 childbearing-age Hispanic individuals (who were either pregnant or non-pregnant) in the Austin area to explore seeking, understanding, and using online information about recommendations for GWG, diet, and physical activity during pregnancy. Using reflexive thematic analysis, we identified both user perspectives and website features that affected the participants’ engagement with and application of online pregnancy information. We conclude that the benefits of online resources fill gaps left by healthcare providers. Nurses as care providers and content creators of health information can help translate guidelines into behaviors that Hispanic people could apply in their everyday lives.

## Introduction

Pregnancy can foster a tremendous need for information and guidance for a healthy lifestyle to support positive birth outcomes. While alcohol consumption and smoking have clear recommendations for abstention, other areas of lifestyle, such as weight gain, diet, and physical activity, are more complex. Thus, women may seek health information to understand which food and nutrient intakes are important to consume during pregnancy, and which should be avoided or consumed in moderation (Chen et al., 2016; Institute of Medicine (US) Committee on Nutritional Status During Pregnancy and Lactation, 1990; March of Dimes, 2025a; Marshall et al., 2022). Similarly, women may also seek information to understand the complexities surrounding physical activity during pregnancy, especially since both encouragement and warnings about activity exist, based on maternal health conditions (American College of Obstetricians and Gynecologists, 2024; Okafor & Goon, 2021).^1^

Another critical dimension of lifestyle during pregnancy is avoiding extremes of inadequate or excessive gestational weight gain (GWG). An astonishing 70% of women gain too little or too much weight during their pregnancy (Branum et al., 2016; Deputy et al., 2015; McElfish et al., 2025; Park et al., 2011; Pawlak et al., 2015), based on guidelines from the Institute of Medicine (Rasmussen & Yaktine, 2009). GWG outside guidelines put pregnant women and their infants at risk for health problems (Goldstein et al., 2017; McElfish et al., 2025; Perumal et al., 2023; Siega-Riz et al., 2009; Viswanathan et al., 2008). Additionally, inadequate GWG increases the risk of preterm birth and low birth weight, whereas excessive GWG increases the risk of cesarean birth, macrosomia, and postpartum weight retention (Goldstein et al., 2017; Perumal et al., 2023; Siega-Riz et al., 2009; Viswanathan et al., 2008). Yet studies indicate that prenatal care providers’ advice about GWG at times may be omitted or incorrect (Arinze et al., 2016; Hollis et al., 2024; Phelan et al., 2011; Waring et al., 2014; Weeks et al., 2018). Consequently, pregnant women often turn to additional sources of health information, such as family, friends, or online resources (Conrad, 2024; Ghiasi, 2021; Huberty et al., 2013; Ledoux et al., 2015).

The Hispanic childbearing population compromises approximately 24% of U.S. births as of 2020 (Osterman et al., 2023) and encounters cultural and other social factors that intersect with lifestyle beliefs and behaviors, which in turn influences GWG (Evenson et al., 2009; Fletcher et al., 2018; Thornton et al., 2006; Tovar et al., 2010). For example, some Hispanic pregnant women believe that food cravings are controlled by the baby’s needs and are beyond their control (Wang et al., 2015). Additionally, Hispanic women have overweight and obesity comparable to Black women or higher than other ethnic groups (National Center for Health Statistics, 2019; Office of Minority Health, 2025). Furthermore, estimates indicate that 25.2% to 29.4% of Hispanic childbearing women gain too little during pregnancy, whereas 41.4% to 42.0% gain too much—rates that are higher than those observed in the overall U.S. childbearing population (Branum et al., 2016; Pawlak et al., 2015; Walker et al., 2014). Hispanic women report seeking pregnancy information from multiple sources such as healthcare providers, nutritionists, or other women (Fletcher et al., 2018; Lindsay et al., 2017; Wang et al., 2015), but the accuracy and consistency of these sources vary (Wang et al., 2015; Weeks et al., 2018). Despite this, only a few studies have examined pregnant Hispanic women’s use of online resources to obtain information on lifestyle concerns during pregnancy (Criss et al., 2015; Lindsay et al., 2017), or how such use may differ from other populations (Chilukuri et al., 2015). This stems in part from the underrepresentation of the Hispanic population in studies (Huberty et al., 2013), exploration of only overall population patterns (Ledoux et al., 2015), or combining diverse minority groups (Mercado et al., 2017). Thus, less is known of Hispanic individuals’ seeking and use of online health information related to GWG, diet, and physical activity during pregnancy.

The overall purpose of this study is to understand the views and experiences in seeking, understanding, and using online health information about recommendations for GWG, diet, and physical activity during pregnancy among Hispanic individuals. Given the importance of pre-pregnancy health behaviors (Wirawan et al., 2023) and the engagement of childbearing-age women with digital health resources for pregnancy preparation (Kostagiolas et al., 2023; Skouteris & Savaglio, 2021; Szwajcer et al., 2005), this study included both pregnant and non-pregnant participants to capture a broader range of perspectives on the use of online pregnancy information. The specific aims were to (1) assess the experience (e.g., usefulness, skills, and barriers encountered) of Hispanic individuals in seeking online information about recommended GWG, diet, and physical activity during pregnancy and (2) determine the views (e.g., usefulness and barriers encountered) of Hispanic individuals about how online recommendations about GWG, diet, and physical activity apply to them in daily living.

## Methods

### Qualitative Descriptive Approach

This is a qualitative descriptive study to explore and understand how the Hispanic childbearing-age population uses online pregnancy information for their health promotion. The qualitative descriptive approach allows researchers to describe a phenomenon of interest by closely elaborating on participants’ perspectives around a phenomenon that is less known about the population (Bradshaw et al., 2017; Sandelowski, 2000, 2010). Thus, this qualitative descriptive approach is the best suited to achieve our study aims to present the population’s direct perspectives and descriptions regarding the use of online pregnancy information.

### Participants and Setting

Childbearing-age Hispanic individuals who had not yet been pregnant (*n* = 10) or who were pregnant (*n* = 10) at the time were recruited using the purposive sampling method (Creswell & Creswell, 2018; Miles et al., 2020). The recruitment was conducted at community centers (a perinatal clinic, children’s wellness center, or an opportunity center) that provide healthcare or other services for underserved populations in Austin between December 2018 and October 2019. Since sample size in a qualitative study cannot be determined before analyzing data (Sandelowski, 1995), prior studies (Lou et al., 2017; Prescott & Mackie, 2017) similar to this project were used as reference to estimate the number of participants (*n* = 20). The staff at the clinics and other sites informed potential participants about the research during their visits, referring those interested to connect with an on-site research team member for further details. For individuals unable to speak with the research team at that time, staff provided them with the study flyer for future reference. The research team identified individuals for eligibility using a screening tool that addressed the following inclusion criteria: (1) pregnant or non-pregnant individuals, (2) self-identified as Latina, Chicana, or Hispanic, for example, Mexican American, (3) between 18 and 44 years, (4) able to read and speak English, and (5) familiar with how to use a computer. Among the diverse labels to describe Latin American descendants, we have chosen to use Hispanic since 90% of the participants identified themselves as either White/Anglo or Mexican American. Latin American descendants who self-identify as either White or Mexican American prefer Hispanic (Martínez & Gonzalez, 2021). Excluded were individuals who self-reported diabetes or other health conditions, which would influence GWG, diet, or physical activity during pregnancy.

### Procedures

Prior to data collection, eligible participants reviewed the informed consent alongside a research team member (S.K. or K.P.) and then signed the informed consent document if they chose to participate. The participants then completed a demographic questionnaire and an established measure of health literacy using the Newest Vital Sign (Pfizer, 2025) to assess participants’ health literacy. If a participant was pregnant during the time of the interview, their pre-pregnancy BMI was estimated using their current weight with a pre-pregnancy weight estimation calculator developed by Thomas et al. (2019) since many participants were unaware of their pre-pregnancy weight. Next, a trained research team member conducted one-on-one interviews for 45 to 60 min in a quiet area at the participating facilities. The participants were randomly assigned one of the following websites: (1) MyPlate | U.S. Department of Agriculture (2025), (2) WebMD (2025), (3) March of Dimes (2025b), (4) BabyCenter (2025), or (5) What to Expect (2025) via a portable electronic device. BabyCenter, WebMD, and What to Expect were selected based on their popularity among pregnant individuals, while USDA MyPlate and March of Dimes were selected due to their reliability as being provided by government or nonprofit websites (Crabbe et al., 2019; Demirci et al., 2016; Snyder et al., 2020). It is important to note that at the time of our study, only one of the five key websites had an option for the Spanish language (BabyCenter). The research team members followed a semi-structured protocol (Table 1) to guide both pregnant and non-pregnant participants in exploring one of the websites for information on GWG, diet, and physical activity. For example, with a website opened, participants were asked *how can you find information about weight gain on this website?* (see Table 1). As the participants attempted to carry out the assigned tasks, they were encouraged to think aloud while searching and exploring information on the website. The researchers took extensive field notes documenting the participants’ search actions and their comments. The interviews were not recorded since some were conducted at a semi-public place. The individuals who participated in this study received a $25 gift card at the end of their interview. This study was approved as exempt by the Institutional Review Board at the University of Texas at Austin (IRB Protocol Number 2017-07-0090).

**Table 1.** Example of Interview Protocol Questions.

1. How can you find information about weight gain on this website?
2. How can you find information about diet on this website?
3. How can you find information about physical activity on this website?
4. What do you think of navigating and understanding the website?
5. What are the confusing/missing information on the website?
6. How would you use the information?
7. What would you like to add more on the website?

### Data Analysis

The team members’ field notes were analyzed via reflexive thematic analysis, recognizing researcher subjectivity as a valuable tool for interpreting and generating meaning (Braun & Clarke, 2006, 2022). Since there was no prior research available on this population, the inductive approach was used to generate new patterns or themes in the participants’ responses regarding using online pregnancy information (Braun & Clarke, 2006, 2022; Vaismoradi et al., 2013). The analysis was conducted by S.K. (a PhD student with an MSN) and L.O.W. (a Professor), both of whom have prior experience analyzing qualitative data in nursing science. Our backgrounds include working with pregnant and postpartum populations in various research contexts. First, to immerse ourselves in the data and familiarize with it, both coders read the field notes multiple times. Second, we started the initial coding process to actively search for meaning by manually highlighting relevant text segments and developing the preliminary codes. Lastly, we reviewed and refined the codes to develop potential themes, iteratively comparing data across participants to establish shared patterns and themes (Thorne, 2000). Both coders remained reflexive throughout the analysis, acknowledging their own positionality in shaping the themes (Braun & Clarke, 2022). These coders regularly discussed the developed codes and themes to resolve discrepancies and ensure coherence within the data. Considering our use of reflexive thematic analysis, data saturation was not a useful criterion for justifying our sample size (Braun & Clarke, 2021; Thorne, 2020; Varpio et al., 2017). Instead, through the iterative process of identifying and comparing data, we generated meaningful insights and patterns that provided sufficient depth to address the study aims and effectively capture participants’ experiences (Braun & Clarke, 2021; Thorne, 2016). The quantitative data related to demographic information and health literacy levels were analyzed using SPSS software (version 28.0.1.1) to describe the participants’ characteristics.

## Results

### Participant Characteristics

Twenty Hispanic individuals (pregnant participant, PP = 10, non-pregnant participant, NPP = 10) participated in this study. Table 2 includes the characteristics of these participants.

**Table 2.** Characteristics of Participants (*n* = 20).

Characteristic	Pregnant participants (*n* = 10)	Non-pregnant participants (*n* = 10)
Mean age (SD^a^, age range)	25.10 (5.49; 18–36)	21.80 (4.44; 18–34)
*Ethnicity*		
White/Anglo	4	
Mexican American	6	8
Central American		1
Cuban		1
*Insurance status*		
Private	3	8
Medicaid	6	
Self-pay	1	2
*Partner status*		
Yes	9	3
No	1	7
*BMI status* ^b,c^		
Normal weight	4	4
Overweight	4	4
Obese	1	2
*Education*		
Partial high school	1	1
High school graduate	3	
Partial college or specialized training	6	6
Standard college or university graduate		2
Graduate professional training		1
*Health literacy status*		
High likelihood of limited literacy	1	
Possibility of limited literacy	1	3
Adequate literacy	8	7
*Gestational age*		Not applicable
1st trimester	1	
2nd trimester	5	
3rd trimester	4	
*Number of children*		Not applicable
0	4	
1	4	
More than 2	2	
*Language preference (speak a language other than English)*		
Never/once in a while	3	
Sometimes	2	2
More than half of the time	1	3
All the time	4	5

*Note*. ^a^SD = standard deviation.

b BMI status: one missing case for pregnant participants.

c Pre-pregnancy BMI status was estimated using pre-pregnancy weight calculator (Thomas et al., 2019).

The mean age of pregnant participants was 25.10 (standard deviation = 5.49) and the mean age of non-pregnant participants was 21.8 (standard deviation = 4.4). Among pregnant participants, 60% were covered under Medicaid, 30% had private insurance, and 10% were self-pay. The majority of non-pregnant participants (80%) had private insurance, while the remaining 20% were self-pay. BMI status between pregnant and non-pregnant groups showed similar distributions (e.g., 40% in normal weight; 40% in overweight). More than half of the participants had completed some college education or specialized training. On the Newest Vital Sign, the majority of participants demonstrated adequate health literacy, defined as scoring over 4 points out of a total of 6 (Pfizer, 2025). Among pregnant participants, 40% were first-time mothers. Language preference of the participants was assessed by whether they speak a language other than English at home, used as an indicator of acculturation (Khan et al., 1997). Nearly half of the participants (45%) reported speaking another language at home all the time, while the remaining 55% reported speaking another language never, once in a while, sometimes, or more than half of the time (see Table 2).

### Seeking Online Information for GWG, Diet, and Physical Activity

The participants demonstrated various approaches when searching the websites for information about weight gain, diet, and physical activity during pregnancy. The five websites differed in layout and organization, which influenced how participants navigated and accessed information. During the searches, participants encountered several factors that impacted both their ability to find information and their understanding of the content presented. The following sub-themes were developed related to information seeking: *discovering/understanding new pregnancy information, engaging with the website features, individuals’ awareness of pregnancy information, needs and interests related to pregnancy*, and *pregnancy related website usability.*

#### Discovering/Understanding New Pregnancy Information

While searching the websites, participants discovered new information related to weight gain, diet, and physical activity during pregnancy. The participants either read web pages or watched short videos to find relevant information for their tasks. Many participants expressed surprise upon discovering new information about what they should or should not do related to diet, exercise, or weight gain during pregnancy. For example, one non-pregnant participant (NPP4) found the article from the What to Expect website, “What to avoid while pregnant,” and stated, “I didn’t know that I cannot eat eggs benedict or hot dogs. I like to have specific examples.” Two other non-pregnant participants (NPP7 and NPP8) discovered that there should not be weight loss during pregnancy. A pregnant participant (PP9) calculated their BMI and found that “My BMI is between 18.5 and 24.9 so I assume 25 – 35 pounds for gestational (weight) gain.” Both pregnant and non-pregnant participants discovered new pregnancy information during their tasks and demonstrated a comprehensive understanding of this new knowledge.

#### Engaging with the Website Features

Participants actively engaged with the interactive website features during their searches. They particularly valued personalized feedback tailored to their individual health information and showed a clear preference for information delivered through interactive or visual formats (e.g., videos or PDF-style brochures) rather than lengthy text. For example, a non-pregnant participant (NPP5) utilized an interactive feature to calculate their BMI based on height and weight, helping them identify an appropriate GWG range. Similarly, a pregnant participant (PP2) noted the usefulness of a BMI chart, stating, “I think it’s good information, including a little chart of BMI ranges and their proper weight gain. My BMI is 30 so. . .. I need to gain 20 pounds during my pregnancy.” Additionally, when videos were available that were relevant to the tasks, the participants showed interest and watched. For instance, a pregnant participant (PP5) liked both videos and written descriptions that provided information on types of exercises and recommended duration. Overall, the different formats and interactive features on the websites provided the participants with an additional way to find and understand information.

#### Individuals’ Awareness of Pregnancy Information

Some participants conducted a search that resulted in general health information, not pregnancy-related information, from the websites. The participants noticed irrelevant information and were able to distinguish it from pregnancy-related information. Both hyperlinks embedded on pregnancy information pages and the participants’ search strategies resulted in retrieving non-pregnancy information. For example, one pregnant participant (PP2) identified a hyperlink labeled “nutrient” on the following article, “Pregnancy Diet: Nutrients You Need,” which led to a page about general nutrient information. The pregnant participant (PP2) stated, “This information is for everybody, not for pregnant women.” While searching for pregnancy exercise information, one non-pregnant participant (NPP6) identified an article titled “What makes you sweat.” After reading the article, this participant (NPP6) recognized that the information was not relevant to pregnancy. In general, participants discerned irrelevant health information during their website search and demonstrated the ability to differentiate between general health information versus pregnancy-related information.

#### Needs and Interests Related to Pregnancy

Websites that reflected childbearing-age individuals’ needs and interests were more effective and engaging in providing pregnancy information to participants. For example, two non-pregnant participants (NPP2 and NPP5) were interested in why they would gain weight during pregnancy. Another non-pregnant participant (NPP4) liked specific food examples related to diet to eat and avoid during pregnancy on the What to Expect website. However, this website did not include an example of a serving size and this participant (NPP4) suggested “I would want an example of what a serving is. . . to make it more usable.” Also, the participant (NPP4) wanted to know more about specific and detailed examples related to safe physical activity during pregnancy such as “I would’ve liked to see more information on if you go to gym, what exercise can you do? If you’re at home, what are some home workouts? What’s best for each trimester?” The other non-pregnant participant (NPP7) liked the website feature from March of Dimes where information started with a question that pregnant individuals would ask. This presentation immediately drew the participant’s attention. When participants found information that aligned with their interests, more time was spent engaging and understanding the information on websites.

Additionally, pregnant participants’ engagement of online information was affected by communication with healthcare providers, as well as gaps in that communication. Specifically, prior knowledge obtained from provider communication impacted pregnant participants’ understanding and application of the pregnancy information gleaned during the study. Not all were informed about healthy GWG by their healthcare providers. For example, when viewing a BMI chart indicating appropriate GWG ranges, a pregnant participant (PP3) realized they were not aware of their BMI; this participant said, “My OB doctor didn’t tell me about my BMI and didn’t tell me about my weight gain during the prenatal visits.” Another pregnant participant (PP4) similarly shared, “My doctor didn’t explain about weight gain. Just told me to eat healthy and don’t eat too much sugar.” A different pregnant participant (PP8) calculated their BMI, discovered they gained more than the recommended GWG for their BMI based on the weight gain chart and said “My doctor didn’t mention it (gestational weight gain). I read lots of online information about weight gain since I didn’t want to gain too much at a certain point.” On the contrary, one pregnant participant (PP9), who does not eat dairy food, was concerned about not getting enough nutrients. This participant had discussed diet with their doctor and was aware of a healthy diet: small frequent meals, recommended calories, and food to avoid during pregnancy. Another participant (PP1) briefly scanned the webpage about healthy weight gain and said “I knew the information on the website. My doctor already told me about it.” Overall, several participants showed a low level of GWG understanding based on communication with their healthcare providers, which impacted their understanding and use of online pregnancy information.

#### Pregnancy Related Website Usability

The five websites utilized in this study had various ways to categorize and present pregnancy information related to GWG, diet, and physical activity, which influenced the participants’ ability to complete their search. Some websites lacked user-centered design, with no dedicated tabs or categories for weight, diet, or physical activity in pregnancy, making navigation difficult. For instance, one pregnant participant (PP2) attempted multiple approaches to find physical activity information on WebMD, but ultimately stated, “At this point, I would Google to find the information that I’m looking for.”

In contrast, participants found information more easily when a “Pregnancy” tab was available in the main menu. For example, a pregnant participant (PP4) quickly accessed relevant content under the “Pregnancy” section on BabyCenter. However, some websites contained outdated, malfunctioning, or misleading hyperlinks. A pregnant participant (PP2), for instance, found a pregnancy nutrition article with a hyperlink that redirected to general healthy diet information. Similarly, a non-pregnant participant (NPP3), who used BabyCenter, noted, “Clicking on one topic took me to a page with multiple hyperlinks for other articles. This was confusing. . . I just think-not that it’s bad, but there are a lot of ads. This makes me really question the site and if it’s accurate.” Additionally, some websites lacked relevant content on GWG, diet, or physical activity, even after participants attempted various search strategies. Others failed to provide clear instructions on how to use interactive features. For example, when a pregnant participant (PP10) tried to calculate their BMI for recommended GWG on What to Expect, they asked the interviewer whether to enter their current or pre-pregnancy weight, as the website provided no guidance.

Overall, websites that were not user-friendly, contained broken or misleading links, lacked relevant content, or failed to provide clear instructions negatively impacted participants’ ability to find and understand pregnancy-related health information.

### Applying Online Recommendations to Daily Living

Several aspects of both the websites and the participants directly influenced the participants’ interactions on the websites and the application of online information related to GWG, diet, and physical activity in their daily lives. The following sub-themes—*usefulness of online pregnancy information* and *applicability of pregnancy website content*—were developed as relevant to the application of pregnancy information found on the websites.

#### Usefulness of Online Pregnancy Information

The participants found the information useful when it provided feasible guidelines to follow in their everyday lives. For instance, the participants thought they could follow and apply the examples of meal plans using recommended healthy ingredients, healthy portion sizes based on common objects, and specific physical activity types and duration. For example, one non-pregnant participant (NPP1) liked the overall layout where options were provided for both “at home” and “at work” physical activities on the USDA MyPlate website. This participant (NPP1) mentioned, “It’s not asking too much of me but would get me moving throughout the day.” Another non-pregnant participant (NPP3), after reading healthy diet information during pregnancy and meal plan examples on the BabyCenter website, commented “I like the examples that are given and I would do that.” Another example of the application of identified information came from a pregnant participant (PP9); this participant liked the content about substitutions or healthier options for when pregnant individuals had food cravings. The pregnant participant (PP9) stated, “I would use this information; healthy frequent snacks and a balanced diet.” On the contrary, one other pregnant participant (PP3) explored information about physical activity during pregnancy on the March of Dimes website and found no actual examples of exercise. This participant (PP3) wanted to know an actual list of exercises that pregnant individuals could do. Additionally, another pregnant participant (PP5) liked the daily calorie intake information that was found on What to Expect. However, this pregnant participant (PP5) wanted to know how much a serving size was for each meal. The participant (PP5) stated “The serving size per day doesn’t give me a specific and feasible serving size. I’m not going to measure my meal with a scale every single time.” When no feasible examples or specific guides were available related to a healthy diet or physical activity during pregnancy, participants showed a struggle to apply the new information to their lives.

#### Applicability of Pregnancy Website Content

Not all of the websites provided the necessary information for the participants to gain a comprehensive understanding of pregnancy related topics such as GWG, diet, and physical activity. This created a gap between finding new information and applying it to their daily lives. For example, a non-pregnant participant (NPP4) found the weight gain information based on BMI for each trimester. However, this non-pregnant participant (NPP4) did not know their own BMI so this participant could not identify a recommended weight gain range during pregnancy. Although the information was available, the participant found it difficult to use due to this missing context. Moreover, some websites contained medical terms or units (e.g., “uterine enlargement,” “mcg,” or “calories”) that participants were not familiar with. Our participants described that information containing these terms or units was not applicable since they could not fully understand the meaning of the information. After reading “How many calories do I need during pregnancy?” on What to Expect, a non-pregnant participant (NPP9) did not like the units that were used to explain calorie amounts and food serving size. Another non-pregnant participant (NPP10) struggled to understand units for daily vitamins presented in a WedMD video about pregnancy supplements. This participant (NPP10) described, “I thought everything is useful information on how much vitamins pregnant women need. But I don’t understand what mcg means.” These unfamiliar terms and incomplete information made it difficult for participants to apply the information to their daily lives.

### Final Interview Questions

At the end of each interview session, participants were asked: (1) Which website they would use or were currently using for pregnancy information, (2) Whether folic acid information was found during their search, and (3) Additional information participants were interested in. All pregnant individuals mainly utilized Google to search for pregnancy information. Some participants shared other pregnancy-related resources such as doctors, pregnancy applications (e.g., Pregnancy+, What to Expect, the BUMP, BabyCenter), or their mothers. While Google was a popular resource among pregnant participants, non-pregnant participants demonstrated more diverse resources: Google, doctors, friends, their mothers, grandmothers, or WebMD. Of the 20 participants, only five found and remembered information about folic acid from their search. Based on the interviewer’s observations, one non-pregnant participant (NPP8) found an article about folic acid but chose not to click on the article. During another non-pregnant participant (NPP10)’s search for a diet, they watched a video on prenatal vitamins, which included folic acid, but they did not recall this afterward. Lastly, participants showed interest in finding additional information on the topics of: breastfeeding, baby-related information (e.g., what is good or bad? baby’s development and how mom’s diet impacts baby’s development?), mental health during pregnancy, and common postpartum issues (such as emotional changes, postpartum depression, and stress).

## Discussion

To our knowledge, this is the first study to examine the usability and usefulness of online health information related to GWG, diet, and physical activity among English-speaking Hispanic pregnant and non-pregnant individuals. Online searching is a prevalent method for obtaining pregnancy-related information (Huberty et al., 2013). This study builds on prior research demonstrating a high level of interest in online health information among pregnant women (Gao et al., 2013; Kavlak et al., 2012; Scaioli et al., 2015) while providing novel insights into the unique experiences of Hispanic childbearing-age individuals. By focusing on this population, the study expands understanding of online health engagement, shedding light on the specific challenges and informational gaps Hispanic pregnant and non-pregnant individuals encounter. It also identifies critical shortcomings in website usability, accessibility, and inclusivity, particularly for individuals with disabilities and non-conforming gender identities.

### User Perspectives

During online information seeking, both pregnant and non-pregnant participants demonstrated discovery and understanding of new pregnancy information regarding GWG, diet, and physical activity, confirming prior findings that online resources supplement unclear or incomplete provider explanations (Huberty et al., 2013; Lagan et al., 2010). The participants particularly valued specific actionable guidelines as well as interactive features such as videos or personalized feedback, supported by Kennedy et al. (2017). According to Kennedy et al. (2017), the pregnant participants were highly interested in applicable features such as recipes, exercise advice, and personalized dietary feedback. Moreover, participants identified misleading or incomplete information and demonstrated varying abilities to evaluate credibility, which is supported by prior findings (Huberty et al., 2013; Lagan et al., 2010).

Contrary to findings in previous studies (Fogel, 2003; Yi et al., 2012), cultural factors did not significantly influence engagement with the websites in this study. The participants’ acculturation status measured by their language preferences (Khan et al., 1997), varied widely—from never to all the time—in speaking a language other than English at home. English-speaking Hispanic individuals in this study were likely more acculturated to mainstream health information sources, which may have reduced their perceived need for culturally tailored content. Additionally, general nutrient-related information, such as healthy ingredients or portion sizes, appeared accessible to all participants. The absence of explicit references to cultural influences may be attributed to the lack of specific prompts regarding cultural preferences in this study, as well as the culturally neutral nature of the online pregnancy information provided.

### Website Content and Usability

Website usability was identified as a critical factor influencing engagement among the childbearing-age population. Issues such as unclear navigation, unfamiliar terms, and malfunctioning features posed barriers to effectively accessing and understanding pregnancy information, even among participants with adequate health literacy. Participants frequently turned to general search engines when credible websites failed to meet their information needs. Choi and Bakken (2010) found similar findings, indicating that participants experienced difficulties navigating websites, encountered incomplete or irrelevant information, and felt overwhelmed by excessive information during the website’s usability test. Additionally, none of the websites reviewed in this study provided inclusive resources for pregnant individuals with disabilities, special needs, or non-conforming gender identities, echoing findings of exclusion in prenatal health resources (Kukura, 2022; Mitra et al., 2016; Moseson et al., 2021). The prenatal environment is a highly gendered space that does not provide much support for gender minority groups (Kukura, 2022; Moseson et al., 2021). Furthermore, Mitra et al. (2016) identified that pregnant women with disabilities shared a lack of information about being pregnant with their disabilities. Pregnant women with disabilities rely heavily on their own online information searches and peer-shared information than guidance from their OB doctors (Mitra et al., 2016). Ruh (2025) underscores the significance of incorporating the perspectives from individuals with disabilities into development and implementation of digital health resources to address barriers and enhance accessibility for populations with diverse physical needs. However, publicly available online resources explored in this study did not provide information tailored to individuals with disabilities and utilized rigid gender terms that spontaneously excluded trans or nonbinary pregnant individuals. Such limitations may discourage the childbearing population with diverse backgrounds from using online pregnancy resources, which can potentially hinder their ability to achieve positive pregnancy health outcomes.

### Communication with Healthcare Providers

Pregnant participants’ engagement with online pregnancy information about GWG and related health behaviors was influenced by their prior communication with healthcare providers. Pregnant participants who had baseline knowledge from previous discussions with healthcare providers engaged more effectively with online information, whereas those without prior guidance struggled to interpret concepts such as pre-pregnancy BMI or estimated GWG. This aligns with previous findings indicating that inconsistent or inadequate GWG discussions during prenatal visits negatively impact women’s understanding (Anderson et al., 2015; Hollis et al., 2024; Weeks et al., 2018). Weeks et al. (2018) identified a wide range of GWG related conversations between pregnant women and healthcare providers. Weeks et al. (2018) also found that GWG information provided by healthcare providers did not follow IOM guidelines and lacked the specifics needed for pregnant women to manage their weight. When healthcare providers did not discuss GWG, pregnant women perceived it as less important (Anderson et al., 2015; Weeks et al., 2018). These findings may explain why some of the participants in this study, without prior GWG knowledge, showed limited engagement with GWG information.

### Theoretical Perspective

Although our themes and sub-themes were inductively developed, many aspects aligned closely with the Uses and Gratifications (U&G) theory (Katz, Blumler, & Gurevitch, 1973; Katz, Haas, & Gurevitch, 1973; McQuail, 1987). According to Katz, Haas, and Gurevitch (1973), people use media to fulfill various needs—such as cognitive, affective, personal integrative, social integrative, and escape dimensions—which lead to gratifications when these needs are met. Given this original model developed in the 1970s, it has some limitations in capturing the multifaceted features of contemporary technologies. Consequently, researchers have adapted the U&G theory to better explain how individuals engage with various new technologies. Sundar and Limperos (2013) introduced Uses and Grats 2.0, an adapted U&G framework tailed to new media that embraces interactive features between users and technologies. This adapted U&G includes four affordances—modality, agency, interactivity, and navigability—that characterize modern electronic technologies and facilitate user gratification. In the current study, the participants’ engagement with pregnancy websites reflected three of these affordances: modality (e.g., engaging with exercise information presented in various formats, such as text or video), interactivity (e.g., calculating recommended GWG using interactive website tools), and navigability (e.g., following hyperlinks access additional information). The agency affordance, defined as users acting as information sources, was not explored due to the scope of this study. Nonetheless, the adapted U&G framework effectively captures various aspects of gratifications that can be achieved through new technologies, and our findings support its applicability in the context of online health information. This adapted U&G illuminates how new digital technologies can enhance user engagement and gratifications (Sundar & Limperos, 2013).

### Strengths and Limitations

In this study, we aimed to understand the use of online pregnancy information related to GWG, diet, and physical activity among Hispanic childbearing-age individuals, who comprise nearly a quarter of those giving birth in the U.S. (Osterman et al., 2023; Tovar et al., 2010). Pregnancy websites hold significant potential to positively contribute to health outcomes in both pregnant individuals and their offspring by delivering critical health information. We found that these pregnancy websites would benefit from improvements that reflect pregnant individuals’ perspectives and needs related to GWG, diet, and physical activity. In doing so, these websites have the potential to be more readily accessible and could enable empowering information for this specific population.

A key strength of this study is the inclusion of both pregnant and non-pregnant individuals, allowing for a broader perspective on the use of pregnancy-related online information. This approach acknowledges that individuals planning for future pregnancies can also benefit from accessible and reliable online health resources. Additionally, this study highlights perspectives of Hispanic individuals of childbearing-age, who are typically under-represented in research despite their rapidly growing birth rates in the U.S. (Osterman et al., 2023). By focusing on their perspectives, this study offers valuable insights into their unique needs and preferences. Particularly, participants provided solid and feasible suggestions on how to improve the utilized websites. These suggestions, if implemented, could be beneficial for the Hispanic childbearing-age population (Table 3).

**Table 3.** Lessons Learned: Guide to Nurses in Digital Health Information Development.

User centered design	
Overall	Consulting with an advisory committee of pregnant people at each stage of the development process
Content	Defining or not using medical or unfamiliar units (e.g., “uterine enlargement,” “mcg,” or “calories”)
	Developing interactive features with literacy adequate instruction (e.g., BMI calculator: include accurate instruction to enter weight before pregnancy and height to obtain BMI and its proper gestational weight gain range)
	Eliminating controversial ads on the websites (e.g., ice-cream ad while reading healthy diet during pregnancy)
	Emphasizing critical information (e.g., folic acid) on the pregnancy main page
	Focusing on possible mental health concerns during pregnancy and postpartum
	Including specific actionable information (e.g., replicable recipes, home exercise options, BMI based monthly weight gain range, healthy diet options)
	Providing comprehensive guideline instead of partial information (e.g., BMI and weight gain range chart without providing a BMI calculator)
	Providing personalized feedback (e.g., weight gain trajectory)
	Using common questions for topics among pregnant people (e.g., why do we gain weight during pregnancy?)
	Utilizing gender inclusive terms and resources for specific populations (e.g., physical activity options for people with selected disabilities)
Navigation	Under pregnancy main tab, listing relevant subcategories
	Using hyperlinks to connect to topic relevant resources

One limitation involves the sample of this study. The participants included a total of 20 English-speaking Hispanic childbearing-age individuals from the Austin area, either pregnant or non-pregnant. This sample is therefore limited by region and language, limiting the generalization of the findings. Another unique limitation is that all participants spoke English, which may reflect higher levels of acculturation to American culture. This could explain why cultural factors were not explicitly discussed in related to online information about GWG, diet, and physical activity. Lastly, the lack of available childcare during interviews may have hindered some participants’ ability to fully engage in study tasks, possibly affecting the completeness of their responses.

### Implications for Nursing Research and Practice

Nurse researchers and clinical nurses can significantly contribute to developing and improving publicly available online health resources by ensuring accurate and guideline-based information on GWG, diet, and physical activity, tailored specifically to needs of pregnant individuals. Furthermore, future research should employ user-centered approaches (Franco et al., 2024; Ruh, 2025) by involving pregnant individuals in designing pregnancy websites and other digital health tools. Adopting these strategies would lessen the burden of navigation among users and improve the dissemination of critical pregnancy-related health information. Supporting these recommendations, participants consistently expressed a preference for information presented in a practical, actionable format that could be easily integrated into their daily lives. For example, Fletcher et al. (2018) reported that Latinas in their study readily forgot numeric GWG guidelines provided by healthcare providers but remembered special behavioral recommendations, such as to “eat no more than one tortilla with each meal” (p. 135). It is important as well that nurses’ efforts should be inclusive of the needs of users with diverse ethnic backgrounds, disabilities, and gender identities to effectively reach the entire childbearing population.

Nurses and other healthcare providers who directly interact with pregnant individuals should identify gaps in the individuals’ understanding of GWG, diet, and physical activity. They should explicitly address these topics during prenatal visits and offer specific, actionable recommendations. Nurses can also guide pregnant individuals toward credible online tools and help them navigate misinformation. Aligning in-person counseling with reliable digital health resources focused on GWG, diet, and physical activity can help improve pregnant individuals’ adherence to the guidelines, ultimately contributing to better maternal and child health outcomes. Our findings support the need to enhance both digital health resources and interpersonal communication between pregnant individuals and healthcare providers. By integrating digital health strategies with clinical practice, nurses can bridge informational gaps. Nurses may also play a critical role as content creators of digital health platforms for pregnant individuals (Bakker et al., 2023; Silva et al., 2022). Insights from this study (see Table 3) support the importance of framing health content in ways that are easily applicable to daily life. The ultimate goal is to ensure that widely available, easy-to-navigate, and inclusive online resources complement nurses’ guidance in disseminating accurate and actionable health information.

In conclusion, this study’s findings highlight the need for more user-centered and culturally responsive digital health tools. By informing research, policy, and digital health initiatives, these insights can contribute to advancing maternal health equity and improving pregnancy-related health outcomes.

## Notes

1. We acknowledge that not all pregnant or potential pregnant individuals identify as women. However, we used “gender terms” when the participants did so in their statements or when we quoted participants were described as women in studies we cited. Otherwise, gender-neutral terms were used to indicate pregnant or non-pregnant participants in this paper.

## References

[bibr1-23333936251334514] American College of Obstetricians and Gynecologists. (2024, September). Exercise during pregnancy. https://www.acog.org/womens-health/faqs/exercise-during-pregnancy

[bibr2-23333936251334514] Anderson C. K. Walch T. J. Lindberg S. M. Smith A. M. Lindheim S. R. Whigham L. D. (2015). Excess gestational weight gain in low-income overweight and obese women: A qualitative study. Journal of Nutrition Education and Behavior, 47(5), 404-411.e1. 10.1016/j.jneb.2015.05.011 PMC4590982 26187348

[bibr3-23333936251334514] Arinze N. V. Karp S. M. Gesell S. B. (2016). Evaluating provider advice and women’s beliefs on total weight gain during pregnancy. Journal of Immigrant and Minority Health, 18(1), 282–286. 10.1007/s10903-015-0162-8 25649967 PMC4715834

[bibr4-23333936251334514] BabyCenter. (2025). Retrieved July 3, 2024, from https://www.babycenter.com/

[bibr5-23333936251334514] Bakker C. J. Wyatt T. H. Breth M. C. Gao G. Janeway L. M. Lee M. A. Martin C. L. Tiase V. L. (2023). Nurses’ roles in mHealth app development: Scoping review. JMIR Nursing, 6, e46058. 10.2196/46058 PMC10618897 37847533

[bibr6-23333936251334514] Bradshaw C. Atkinson S. Doody O. (2017). Employing a qualitative description approach in health care research. Global Qualitative Nursing Research, 4, 1–8. 10.1177/2333393617742282 PMC5703087 29204457

[bibr7-23333936251334514] Branum A. M. Sharma A. J. Deputy N. P. (2016). QuickStats: Gestational weight gain among women with full-term, singleton births, compared with recommendations—48 States and the District of Columbia, 2015. MMWR. Morbidity and Mortality Weekly Report, 65, 1121. 10.15585/mmwr.mm6540a10 27736838

[bibr8-23333936251334514] Braun V. Clarke V. (2006). Using thematic analysis in psychology. Qualitative Research in Psychology, 3(2), 77–101. 10.1191/1478088706qp063oa

[bibr9-23333936251334514] Braun V. Clarke V. (2021). To saturate or not to saturate? Questioning data saturation as a useful concept for thematic analysis and sample-size rationales. Qualitative Research in Sport, Exercise and Health, 13(2), 201–216. 10.1080/2159676X.2019.1704846

[bibr10-23333936251334514] Braun V. Clarke V. (2022). Thematic analysis: A practical guide. Sage.

[bibr11-23333936251334514] Chen X. Zhao D. Mao X. Xia Y. Baker P. Zhang H. (2016). Maternal dietary patterns and pregnancy outcome. Nutrients, 8(6), 351. 10.3390/nu8060351 27338455 PMC4924192

[bibr12-23333936251334514] Chilukuri N. West M. Henderson J. L. Lawson S. Ehsanipoor R. Costigan K. Polk S. Bennett W. (2015). Information and communication technology use among low-income pregnant and postpartum women by race and ethnicity: A cross-sectional study. Journal of Medical Internet Research, 17(7), e163. 10.2196/jmir.3916 PMC4526977 26142162

[bibr13-23333936251334514] Choi J. Bakken S. (2010). Web-based education for low-literate parents in Neonatal Intensive Care Unit: Development of a website and heuristic evaluation and usability testing. International Journal of Medical Informatics, 79(8), 565–575. 10.1016/j.ijmedinf.2010.05.001 20617546 PMC2956000

[bibr14-23333936251334514] Conrad M. (2024). Health information-seeking internet behaviours among pregnant women: A narrative literature review. Journal of Reproductive and Infant Psychology, 42(2), 194–208. 10.1080/02646838.2022.2088711 35703164

[bibr15-23333936251334514] Crabbe R. E. S. Stone P. Filoche S. K. (2019). What are women saying about noninvasive prenatal testing? An analysis of online pregnancy discussion forums. Prenatal Diagnosis, 39(10), 890–895. 10.1002/pd.5500 31172546

[bibr16-23333936251334514] Creswell J. W. Creswell J. D. (2018). Research design: Qualitative, quantitative, and mixed methods approaches (5th ed.). Sage.

[bibr17-23333936251334514] Criss S. Woo Baidal J. A. Goldman R. E. Perkins M. Cunningham C. Taveras E. M. (2015). The role of health information sources in decision-making among Hispanic mothers during their children’s first 1000 days of life. Maternal and Child Health Journal, 19(11), 2536–2543. 10.1007/s10995-015-1774-2 26122256 PMC4596758

[bibr18-23333936251334514] Demirci J. R. Cohen S. M. Parker M. Holmes A. Bogen D. L. (2016). Access, use, and preferences for technology-based perinatal and breastfeeding support among childbearing women. The Journal of Perinatal Education, 25(1), 29–36. 10.1891/1058-1243.25.1.29 26848248 PMC4719111

[bibr19-23333936251334514] Deputy N. P. Sharma A. J. Kim S. Y. Hinkle S. N. (2015). Prevalence and characteristics associated with gestational weight gain adequacy. Obstetrics & Gynecology, 125(4), 773. 10.1097/AOG.0000000000000739 25751216 PMC4425284

[bibr20-23333936251334514] Evenson K. R. Moos M. K. Carrier K. Siega-Riz A. M. (2009). Perceived barriers to physical activity among pregnant women. Maternal and Child Health Journal, 13(3), 364–375. 10.1007/s10995-008-0359-8 18478322 PMC2657195

[bibr21-23333936251334514] Fletcher G. E. Teeters L. Schlundt D. Bonnet K. Heerman W. J. (2018). Maternal conception of gestational weight gain among Latinas: A qualitative study. Health Psychology, 37(2), 132–138. 10.1037/hea0000555 28967775 PMC5794623

[bibr22-23333936251334514] Fogel J. (2003). Internet use for cancer information among racial/ethnic populations and low literacy groups. Cancer Control, 10(5 Suppl), 45–51. 10.1177/107327480301005s07 14581904

[bibr23-23333936251334514] Franco P. Olhaberry M. Muzard A. Harismendy Á. Kelders S. (2024). Developing a guided web app for postpartum depression symptoms: User-centered design approach. JMIR Formative Research, 8, e56319. 10.2196/56319 PMC11369531 39159447

[bibr24-23333936251334514] Gao L. Larsson M. Luo S. (2013). Internet use by Chinese women seeking pregnancy-related information. Midwifery, 29(7), 730–735. 10.1016/j.midw.2012.07.003 22958935

[bibr25-23333936251334514] Ghiasi A. (2021). Health information needs, sources of information, and barriers to accessing health information among pregnant women: A systematic review of research. The Journal of Maternal-Fetal & Neonatal Medicine, 34(8), 1320–1330. 10.1080/14767058.2019.1634685 31216921

[bibr26-23333936251334514] Goldstein R. F. Abell S. K. Ranasinha S. Misso M. Boyle J. A. Black M. H. Li N. Hu G. Corrado F. Rode L. Kim Y. J. Haugen M. Song W. O. Kim M. H. Bogaerts A. Devlieger R. Chung J. H. Teede H. J. (2017). Association of gestational weight gain with maternal and infant outcomes: A systematic review and meta-analysis. JAMA, 317(21), 2207. 10.1001/jama.2017.3635 28586887 PMC5815056

[bibr27-23333936251334514] Hollis J. L. Deroover K. Licata M. Tully B. Farragher E. Lecathelinais C. Bennett N. Foster M. Pennell C. E. Wiggers J. Daly J. Kingsland M. (2024). Antenatal care addressing gestational weight gain (GWG): A cross sectional study of pregnant women’s reported receipt and acceptability of recommended GWG care and associated characteristics. BMC Pregnancy and Childbirth, 24(1), 111. 10.1186/s12884-023-06158-4 38321389 PMC10845753

[bibr28-23333936251334514] Huberty J. Dinkel D. Beets M. W. Coleman J. (2013). Describing the use of the Internet for health, physical activity, and nutrition information in pregnant women. Maternal and Child Health Journal, 17(8), 1363–1372. 10.1007/s10995-012-1160-2 23090284

[bibr29-23333936251334514] Institute of Medicine (US) Committee on Nutritional Status During Pregnancy and Lactation. (1990). Nutrition During Pregnancy: Part I Weight Gain: Part II Nutrient Supplements. National Academies Press (US). http://www.ncbi.nlm.nih.gov/books/NBK235228/25144018

[bibr30-23333936251334514] Katz E. Blumler J. G. Gurevitch M. (1973). Uses and gratifications research. The Public Opinion Quarterly, 37(4), 509–523.

[bibr31-23333936251334514] Katz E. Haas H. Gurevitch M. (1973). On the use of the mass media for important things. American Sociological Review, 38(2), 164–181. 10.2307/2094393

[bibr32-23333936251334514] Kavlak O. Atan Ü. Güleç D. Öztürk R. Atay N. (2012). Pregnant women’s use of the internet in relation to their pregnancy in Izmir, Turkey. Informatics for Health & Social Care, 37(4), 253–263. 10.3109/17538157.2012.710686 22958087

[bibr33-23333936251334514] Kennedy R. A. K. Mullaney L. Reynolds C. M. E. Cawley S. McCartney D. M. A. Turner M. J. (2017). Preferences of women for web-based nutritional information in pregnancy. Public Health, 143, 71–77. 10.1016/j.puhe.2016.10.028 28159029

[bibr34-23333936251334514] Khan L. K. Sobal J. Martorell R. (1997). Acculturation, socioeconomic status, and obesity in Mexican Americans, Cuban Americans, and Puerto Ricans. International Journal of Obesity, 21(2), 91-96. 10.1038/sj.ijo.0800367 9043961

[bibr35-23333936251334514] Kostagiolas P. Alexopoulou E. Ntalampiras S. Stamou S. Theodorou P. Niakas D. (2023). Pregnancy-related information-seeking behavior and information literacy profiles of women at reproductive age. Journal of Hospital Librarianship, 23(4), 242–263. 10.1080/15323269.2023.2262933

[bibr36-23333936251334514] Kukura E. (2022). Reconceiving reproductive health systems: Caring for trans, nonbinary, and gender-expansive people during pregnancy and childbirth. Journal of Law, Medicine & Ethics, 50(3), 471–488. 10.1017/jme.2022.88 PMC9679586 36398635

[bibr37-23333936251334514] Lagan B. M. Sinclair M. Kernohan W. G. (2010). Internet use in pregnancy informs women’s decision making: A web-based survey. Birth, 37(2), 106–115. 10.1111/j.1523-536X.2010.00390.x 20557533

[bibr38-23333936251334514] Ledoux T. Van Den Berg P. Leung P. Berens P. D. (2015). Factors associated with knowledge of personal gestational weight gain recommendations. BMC Research Notes, 8, 1–7. 10.1186/s13104-015-1306-6 26268578 PMC4534067

[bibr39-23333936251334514] Lindsay A. C. Wallington S. F. Greaney M. L. Tavares Machado M. M. De Andrade G. P. (2017). Patient-provider communication and counseling about gestational weight gain and physical activity: A qualitative study of the perceptions and experiences of Latinas pregnant with their first child. International Journal of Environmental Research and Public Health, 14(11), 1412. 10.3390/ijerph14111412 29156548 PMC5708051

[bibr40-23333936251334514] Lou S. Frumer M. Schlütter M. M. Petersen O. B. Vogel I. Nielsen C. P. (2017). Experiences and expectations in the first trimester of pregnancy: A qualitative study. Health Expectations : An International Journal of Public Participation in Health Care and Health Policy, 20(6), 1320–1329. 10.1111/hex.12572 28521069 PMC5689234

[bibr41-23333936251334514] March of Dimes. (2025a). Exercise during pregnancy. https://www.marchofdimes.org/find-support/topics/pregnancy/exercise-during-pregnancy

[bibr42-23333936251334514] March of Dimes. (2025b). Retrieved July 3, 2024, from https://www.marchofdimes.org/

[bibr43-23333936251334514] Marshall N. E. Abrams B. Barbour L. A. Catalano P. Christian P. Friedman J. E. Hay W. W. Hernandez T. L. Krebs N. F. Oken E. Purnell J. Q. Roberts J. M. Soltani H. Wallace J. Thornburg K. L. (2022). The importance of nutrition in pregnancy and lactation: Lifelong consequences. American Journal of Obstetrics and Gynecology, 226(5), 607–632. 10.1016/j.ajog.2021.12.035 34968458 PMC9182711

[bibr44-23333936251334514] Martínez D. E. Gonzalez K. E. (2021). “Latino” or “Hispanic”? The sociodemographic correlates of panethnic label preferences among U.S. Latinos/Hispanics. Sociological Perspectives, 64(3), 365–386. 10.1177/0731121420950371

[bibr45-23333936251334514] McElfish P. A. Ayers B. L. Hawley N. L. Caldwell A. Porter A. Macechko M. D. Watson D. Callaghan-Koru J. A. Selig J. P. Andersen J. A. Manning N. White L. Gomez-Pomar E. Brown C. C. (2025). Gestational weight gain and increased risk of cesarean delivery across body mass index categories. AJOG Global Reports, 5(1), 100445. 10.1016/j.xagr.2025.100445 40027475 PMC11869019

[bibr46-23333936251334514] McQuail D. (1987). Mass communication theory: An introduction. (2nd ed.). Sage.

[bibr47-23333936251334514] Mercado A. Marquez B. Abrams B. Phipps M. G. Wing R. R. Phelan S. (2017). Where do women get advice about weight, eating, and physical activity during pregnancy? Journal of Women’s Health, 26(9), 951–956. 10.1089/jwh.2016.6078 PMC5649418 28525293

[bibr48-23333936251334514] Miles M. B. Huberman A. M. Saldaña J. (2020). Qualitative data analysis: A methods sourcebook. (4th ed.). Sage.

[bibr49-23333936251334514] Mitra M. Long-Bellil L. M. Iezzoni L. I. Smeltzer S. C. Smith L. D. (2016). Pregnancy among women with physical disabilities: Unmet needs and recommendations on navigating pregnancy. Disability and Health Journal, 9(3), 457–463. 10.1016/j.dhjo.2015.12.007 26847669 PMC4903955

[bibr50-23333936251334514] Moseson H. Fix L. Hastings J. Stoeffler A. Lunn M. R. Flentje A. Lubensky M. E. Capriotti M. R. Ragosta S. Forsberg H. Obedin-Maliver J. (2021). Pregnancy intentions and outcomes among transgender, nonbinary, and gender-expansive people assigned female or intersex at birth in the United States: Results from a national, quantitative survey. International Journal of Transgender Health, 22(1–2), 30–41. 10.1080/26895269.2020.1841058 34796363 PMC8040680

[bibr51-23333936251334514] MyPlate | U.S. Department of Agriculture. (2025). Retrieved July 3, 2024, from https://www.myplate.gov/

[bibr52-23333936251334514] National Center for Health Statistics. (2019). Table 26. Normal weight, overweight, and obesity among adults aged 20 and over, by selected characteristics: United States, selected years 1988–1994 through 2015–2018. U.S. Centers for Disease Control and Prevention. https://www.cdc.gov/nchs/data/hus/2019/026-508.pdf

[bibr53-23333936251334514] Office of Minority Health. (2025, Feb 13). Obesity and Hispanic Americans. U.S. Department of Health & Human Services. https://minorityhealth.hhs.gov/obesity-and-hispanic-americans

[bibr54-23333936251334514] Okafor U. B. Goon D. T. (2021). Physical activity in pregnancy: Beliefs, benefits, and information-seeking practices of pregnant women in South Africa. Journal of Multidisciplinary Healthcare, 14, 787–798. 10.2147/JMDH.S287109 33859477 PMC8043848

[bibr55-23333936251334514] Osterman M. J. K. Hamilton B. E. Martin J. A. Driscoll A. K. Valenzuela C. P. (2023). Births: Final Data for 2021. National Vital Statistics Reports: From the Centers for Disease Control and Prevention, National Center for Health Statistics, National Vital Statistics System, 72(1), 1–53. 36723449

[bibr56-23333936251334514] Park S. Sappenfield W. M. Bish C. Salihu H. Goodman D. Bensyl D. M. (2011). Assessment of the Institute of Medicine recommendations for weight gain during pregnancy: Florida, 2004–2007. Maternal and Child Health Journal, 15(3), 289–301. 10.1007/s10995-010-0596-5 20306221

[bibr57-23333936251334514] Pawlak M. T. Alvarez B. T. Jones D. M. Lezotte D. C. (2015). The effect of race/ethnicity on gestational weight gain. Journal of Immigrant and Minority Health, 17(2), 325–332. 10.1007/s10903-013-9886-5 23934517

[bibr58-23333936251334514] Perumal N. Wang D. Darling A. M. Liu E. Wang M. Ahmed T. Christian P. Dewey K. G. Kac G. Kennedy S. H. Subramoney V. Briggs B. Fawzi W. W. (2023). Suboptimal gestational weight gain and neonatal outcomes in low and middle income countries: Individual participant data meta-analysis. BMJ, 382, e072249. 10.1136/bmj-2022-072249 PMC10512803 37734757

[bibr59-23333936251334514] Pfizer. (2025). The Newest Vital Sign. Retrieved July 3, 2024, from https://www.pfizer.com/products/medicine-safety/health-literacy/nvs-toolkit

[bibr60-23333936251334514] Phelan S. Phipps M. G. Abrams B. Darroch F. Schaffner A. Wing R. R. (2011). Practitioner advice and gestational weight gain. Journal of Women’s Health, 20(4), 585–591. 10.1089/jwh.2010.2316 PMC3075048 21413898

[bibr61-23333936251334514] Prescott J. Mackie L. (2017). “You sort of go down a rabbit hole. . .You’re just going to keep on searching”: A qualitative study of searching online for pregnancy-related information during pregnancy. Journal of Medical Internet Research, 19(6), e194–e194. 10.2196/jmir.6302 PMC5476867 28583906

[bibr62-23333936251334514] Rasmussen K. M. Yaktine A. L. (Eds.). (2009). Weight gain during pregnancy: Reexamining the guidelines. The National Academies Press. 20669500

[bibr63-23333936251334514] Ruh D. M. (2025). Patient perspectives on digital health. In Patel D. (Ed.), Digital health (pp. 481–501). Academic Press. 10.1016/B978-0-443-23901-4.00035-0

[bibr64-23333936251334514] Sandelowski M. (1995). Sample size in qualitative research. Research in Nursing & Health, 18(2), 179–183. 10.1002/nur.4770180211 7899572

[bibr65-23333936251334514] Sandelowski M. (2000). Whatever happened to qualitative description? Research in Nursing & Health, 23(4), 334–340. 10.1002/1098-240X(200008)23:4<334::AID-NUR9>3.0.CO;2-G 10940958

[bibr66-23333936251334514] Sandelowski M. (2010). What’s in a name? Qualitative description revisited. Research in Nursing & Health, 33(1), 77–84. 10.1002/nur.20362 20014004

[bibr67-23333936251334514] Scaioli G. Bert F. Galis V. Brusaferro S. De Vito E. La Torre G. Manzoli L. Messina G. Torregrossa M. V. Ricciardi W. Gualano M. R. Siliquini R. (2015). Pregnancy and internet: Sociodemographic and geographic differences in e-health practice. Results from an Italian multicenter study. Public Health, 129(9), 1258–1266. 10.1016/j.puhe.2015.06.012 26210071

[bibr68-23333936251334514] Siega-Riz A. M. Viswanathan M. Moos M. K. Deierlein A. Mumford S. Knaack J. Thieda P. Lux L. J. Lohr K. N. (2009). A systematic review of outcomes of maternal weight gain according to the Institute of Medicine recommendations: Birthweight, fetal growth, and postpartum weight retention. American Journal of Obstetrics and Gynecology, 201(4), 339.e1-339.e14. 10.1016/j.ajog.2009.07.002 19788965

[bibr69-23333936251334514] Silva L. D. da Bar K. A. Zamberlan A. O. Ben L. W. D. Sasso G. M. D. Backes D. S. (2022). Web app for the monitoring of pregnant and puerperal women: Technological production. Online Brazilian Journal of Nursing, 21(1), 1–14. 10.17665/1676-4285.20226529

[bibr70-23333936251334514] Skouteris H. Savaglio M. (2021). The use of social media for preconception information and pregnancy planning among young women. Journal of Clinical Medicine, 10(9), 1892. 10.3390/jcm10091892 33925520 PMC8123806

[bibr71-23333936251334514] Snyder A. Neufeld H. T. Forbes L. (2020). A mixed-methods investigation of women’s experiences seeking pregnancy-related online nutrition information. BMC Pregnancy and Childbirth, 20(1), 377. 10.1186/s12884-020-03065-w 32590955 PMC7320538

[bibr72-23333936251334514] Sundar S. S. Limperos A. M. (2013). Uses and Grats 2.0: New gratifications for new media. Journal of Broadcasting & Electronic Media, 57(4), 504–525. 10.1080/08838151.2013.845827

[bibr73-23333936251334514] Szwajcer E. M. Hiddink G. J. Koelen M. A. Van Woerkum C. M. J. (2005). Nutrition-related information-seeking behaviours before and throughout the course of pregnancy: Consequences for nutrition communication. European Journal of Clinical Nutrition, 59(S1), S57–S65. 10.1038/sj.ejcn.1602175 16052197

[bibr74-23333936251334514] Thomas D. M. Oken E. Rifas-Shiman S. L. Téllez-Rojo M. Just A. Svensson K. Deierlein A. L. Chandler-Laney P. C. Miller R. C. McNamara C. Phelan S. Yoshitani S. Butte N. F. Redman L. M. (2019). Do women know their prepregnancy weight? Obesity, 1161-1167. 10.1002/oby.22502 PMC6834084 31149770

[bibr75-23333936251334514] Thorne S. E. (2000). Data analysis in qualitative research. Evidence-Based Nursing, 3(3), 68-70. 10.1136/ebn.3.3.68

[bibr76-23333936251334514] Thorne S. E. (2016). Interpretive description: Qualitative research for applied practice (2nd ed.). Routledge.

[bibr77-23333936251334514] Thorne S. E. (2020). The great saturation debate: What the “S Word” means and doesn’t mean in qualitative research reporting. Canadian Journal of Nursing Research, 52(1), 3–5. 10.1177/0844562119898554 31928347

[bibr78-23333936251334514] Thornton P. L. Kieffer E. C. Salabarría-Peña Y. Odoms-Young A. Willis S. K. Kim H. Salinas M. A. (2006). Weight, diet, and physical activity-related beliefs and practices among pregnant and postpartum Latino women: The role of social support. Maternal and Child Health Journal, 10(1), 95–104. 10.1007/s10995-005-0025-3 16534660

[bibr79-23333936251334514] Tovar A. Chasan-Taber L. Bermudez O. I. Hyatt R. R. Must A. (2010). Knowledge, attitudes, and beliefs regarding weight gain during pregnancy among Hispanic women. Maternal and Child Health Journal, 14(6), 938–949. 10.1007/s10995-009-0524-8 19760160 PMC3278222

[bibr80-23333936251334514] Vaismoradi M. Turunen H. Bondas T. (2013). Content analysis and thematic analysis: Implications for conducting a qualitative descriptive study. Nursing & Health Sciences, 15(3), 398–405. 10.1111/nhs.12048 23480423

[bibr81-23333936251334514] Varpio L. Ajjawi R. Monrouxe L. V. O’Brien B. C. Rees C. E. (2017). Shedding the cobra effect: Problematising thematic emergence, triangulation, saturation and member checking. Medical Education, 51(1), 40–50. 10.1111/medu.13124 27981658

[bibr82-23333936251334514] Viswanathan M. Siega-Riz A. M. Moos M. K. Deierlein A. Mumford S. Knaack J. Thieda P. Lux L. J. Lohr K. N. (2008). Outcomes of maternal weight gain. Agency for Healthcare Research and Quality. PMC4781425 18620471

[bibr83-23333936251334514] Walker L. O. Cheng H. R. Brown A. (2014). Birth outcomes of Hispanic women and risks or strengths associated with ethnicity and Texas border residence. Journal of Obstetric, Gynecologic, & Neonatal Nursing, 43(4), 422–434. 10.1111/1552-6909.12467 24947021

[bibr84-23333936251334514] Wang M. L. Arroyo J. Druker S. Sankey H. Z. Rosal M. C. (2015). Knowledge, attitudes and provider advice by pre-pregnancy weight status: A qualitative study of pregnant Latinas with excessive gestational weight gain. Women & Health, 55(7), 805–828. 10.1080/03630242.2015.1050542 26016948

[bibr85-23333936251334514] Waring M. E. Moore Simas T. A. Barnes K. C. Terk D. Baran I. Pagoto S. L. Rosal M. C. (2014). Patient report of guideline-congruent gestational weight gain advice from prenatal care providers: Differences by prepregnancy BMI. Birth, 41(4), 353–359. 10.1111/birt.12131 25187296

[bibr86-23333936251334514] WebMD. (2025). Retrieved July 3, 2024, from https://www.webmd.com/

[bibr87-23333936251334514] Weeks A. Liu R. H. Ferraro Z. M. Deonandan R. Adamo K. B. (2018). Inconsistent weight communication among prenatal healthcare providers and patients: A narrative review. Obstetrical & Gynecological Survey, 73(8), 486–499. 10.1097/OGX.0000000000000588 30169887

[bibr88-23333936251334514] What to Expect. (2025). Retrieved July 3, 2024, from https://www.whattoexpect.com/

[bibr89-23333936251334514] Wirawan F. Yudhantari D. G. A. Gayatri A. (2023). Pre-pregnancy diet to maternal and child health outcome: A scoping review of current evidence. Journal of Preventive Medicine and Public Health, 56(2), 111–127. 10.3961/jpmph.22.472 37055354 PMC10111095

[bibr90-23333936251334514] Yi Y. J. Stvilia B. Mon L. (2012). Cultural influences on seeking quality health information: An exploratory study of the Korean community. Library & Information Science Research, 34(1), 45–51. 10.1016/j.lisr.2011.06.001

